# The Roles of Fractionated Potentials in Non-Macroreentrant Atrial Tachycardias Following Atrial Fibrillation Ablation: Recognition Beyond Three-Dimensional Mapping

**DOI:** 10.3389/fcvm.2021.759563

**Published:** 2022-03-10

**Authors:** Yu-Chuan Wang, Li-Bin Shi, Song-Yun Chu, Eivind Solheim, Peter Schuster, Jian Chen

**Affiliations:** ^1^Department of Geriatrics, Peking University First Hospital, Beijing, China; ^2^Department of Heart Disease, Haukeland University Hospital, Bergen, Norway; ^3^Department of Clinical Science, University of Bergen, Bergen, Norway; ^4^Department of Cardiology, Peking University First Hospital, Beijing, China

**Keywords:** atrial tachycardia, atrial fibrillation, fractionated potential, mapping, ablation

## Abstract

**Introduction:**

Non-macroreentrant atrial tachycardia (nAT) following atrial fibrillation (AF) ablation is being increasingly reported. Many issues remain to be elucidated. We aimed to characterize the fractionated potentials (FPs) in nAT and introduce a new method of cross-mapping for clarifying their roles.

**Methods and Results:**

Forty-four nATs in 37 patients were enrolled and classified into focal AT (FAT, 12), microreentrant AT (MAT, 14), and small-loop-reentrant AT (SAT, 18) groups, according to activation pattern. FP was found on all targets except in nine FATs. The ratio of FP duration to AT cycle length (TCL) was different among groups (28 ± 7% in FAT, 53 ± 11% in MAT, and 42 ± 14% in SAT, *p* < 0.05), and ablation duration were longer in SATs (313 ± 298 vs. 111 ± 125 s, *p* < 0.05). The ratio of mappable cycle length to TCL was lower in the FAT group (63 ± 22% vs. 90 ± 9% and 89 ± 8%, *p* < 0.05). When cross-mapping was employed, trans-potential time differences in both longitudinal and transverse direction were longer around the culprit FP for MAT (*p* < 0.01). After Receiver Operating Characteristic curve analysis, it is best to adopt the sum of time difference ratios in both directions ≥60% as a cut-off value for discrimination of the FPs responsible for MAT with a sensitivity of 92% and specificity of 87%.

**Conclusions:**

FP could be found on target in most nATs following a previous AF ablation. The ratio of FP duration to TCL may help for differentiation. A simple method of cross-mapping could be employed to clarify the roles of FPs.

## Introduction

Catheter ablation has become an important therapy for atrial fibrillation (AF). Pulmonary vein isolation is a cornerstone for AF ablation technique (1). Expansive ablation techniques, including linear ablation and complex fractionated atrial electrogram (CFAE) ablation, have been employed to improve clinical outcomes of ablation for persistent AF (2, 3). As a consequence, atrial tachycardia (AT) post AF ablation has been increasingly observed. Compared with the incidence of <5% after segmental pulmonary vein ablation (4, 5), the incidence increases to 10–31% after circumferential pulmonary vein ablation (6, 7), to >20% after CFAE ablation (8), and to nearly 50% after an extensive stepwise approach of AF ablation (9). Among the post AF ablation ATs, 25% was accounted for non-macroreentrant ATs (nATs), with almost 75% for macroreentrant ATs, whose reentry circuit diameter was ≥30 mm and involved more than two parts of the atria (6, 10). In patients with post AF ablation AT, symptoms are often significant due to a rapid and regular ventricular response and are refractory to antiarrhythmic drugs. Therefore, catheter ablation has been playing an increasingly important role for these ATs. Meanwhile, great challenges of procedures are presented by the complexity, variability, and unpredictability of these ATs, especially in nATs (11). Although nATs have been reported, many issues have not yet been elucidated. In this study, we systematically investigated and compared the electrophysiological characteristics of different nATs.

## Methods

### Study Population

Patients with AT were retrospectively enrolled when the following criteria were satisfied: (i) There was clinical evidence for AT on surface electrocardiogram or/and Holter and diagnoses were confirmed by electrophysiological study; (ii) patients had undergone at least one ablation procedure for AF prior to AT ablation; (iii) AT was successfully eliminated by ablation during the procedure. All patients provided informed consent before the ablation procedure. The study protocol was approved by the Ethics Committee of Western Norway.

### Electrophysiological Study and Ablation

**Figure 1:**
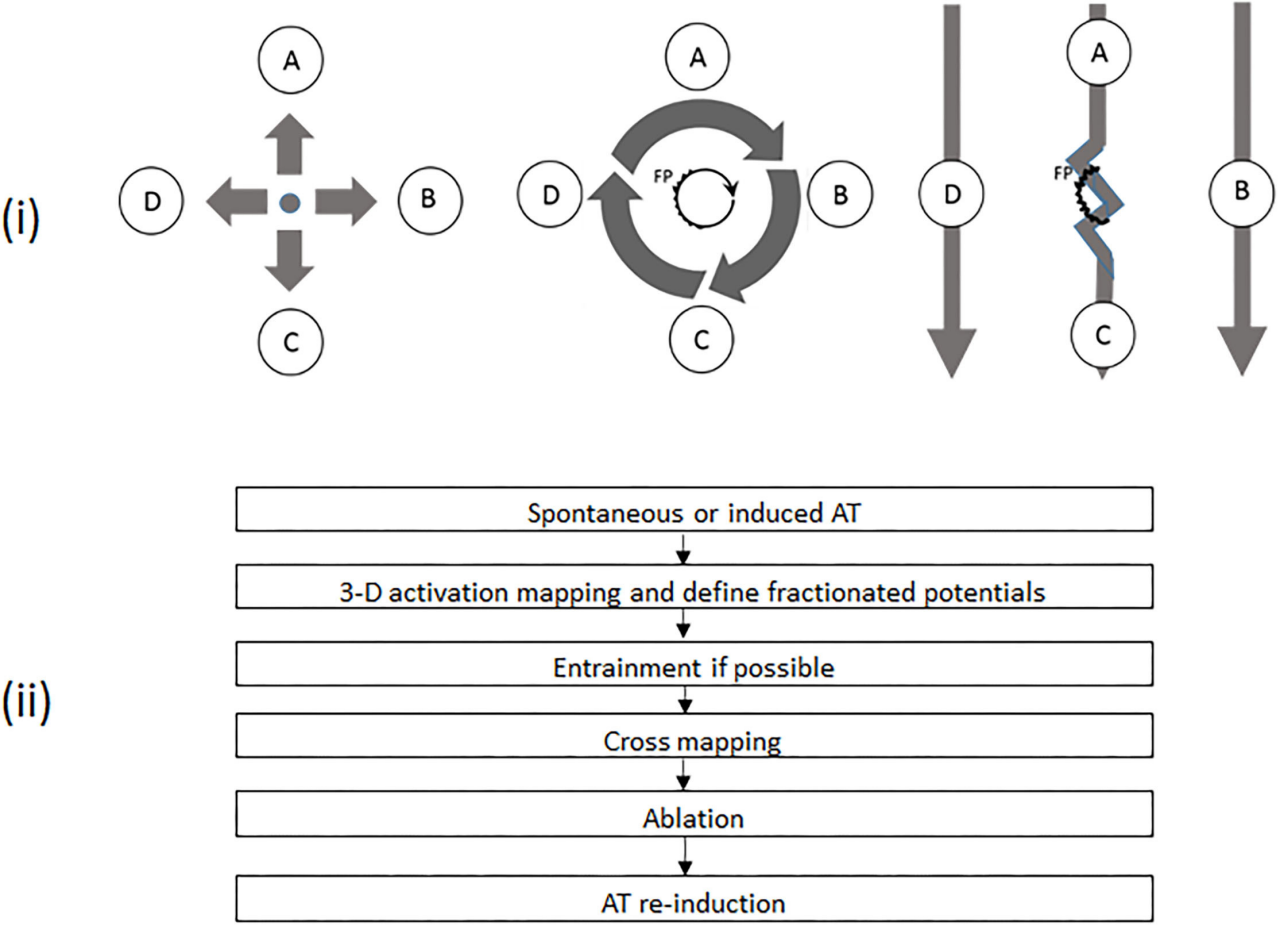
(i) Schematic diagrams demonstrating different activation patterns in various atrial tachycardias (ATs) and the principles of cross-mapping for the differentiation of different ATs with fractionated potentials (FPs). The left panel shows a focal AT with sites A, B, C, and D activated almost at the same time. The middle panel demonstrates a microreentrant AT, with sites A, B, C, and D activated sequentially. The core with a FP is the source for maintenance of tachycardia. In the right panel, FP is a bystander potential. Thus, Sites A and C are activated sequentially due to slow conduction through FP. Sites B and D are activated almost simultaneously. The twisted line represents an area with FP. The heavy arrow shows the direction of activation. A, B, C, and D represent mapping sites around an FP, respectively. (ii) Workflow chart of mapping and ablation. AT, atrial tachycardia; FP, fractionated potential.

All antiarrhythmic medications, except for amiodarone, were discontinued for at least five half-lives. All procedures were performed under conscious sedation. A 20-pole catheter (Abbott, Minneapolis, MN, USA) with 2–10–2 mm spacing was advanced into the high right atrium (RA) and looped around the tricuspid annulus with the distal dipoles in the coronary sinus (CS). After a single transseptal puncture, an ablation catheter (Thermocool, Biosense Webster, Diamond Bar, CA, USA or Therapy Cool Path, Abbott, Minneapolis, MN, USA) and a circular mapping catheter (Lasso, Biosense Webster or Inquiry Optima, Abbott) were advanced into the left atrium (LA). The procedure was performed step by step as follows: (i) AT was induced by electrical stimulation if sinus rhythm presented before the procedure; (ii) three-dimensional activation mapping was performed by means of CARTO (Biosense Webster) or EnSite NavX system (Abbott); (iii) entrainment maneuvers were employed to identify the mechanism of AT, if possible, but was not performed in patients for whom there was concern regarding AT converting to another form or degenerating into AF; (iv) as a complement, a new method of cross-mapping was used at areas of interest to differentiate diagnoses when entrainment could not be applied or produced an unsatisfactory result; (v) radiofrequency energy was delivered at the identified essential locations; (vi) electrical stimulation was implemented for re-induction after the termination of AT by ablation (Figure 1).

After overall activation mapping, the areas of interest, including suspicious reentrant circuits and fractionated potential (FP), were meticulously mapped with a distance between points ≤5 mm. FP was defined as a multi-component electrogram with ≥3 deflections (12, 13). When an FP was recorded, cross-mapping with four recording sites evenly and closely distributed (pairwise facing and vertical to each other) around the FP (Figure 1) was used to identify the possible role of FP in tachycardia. The time difference was measured across the FP in two perpendicular directions. The direction with the longer time difference was defined as longitudinal, and the shorter as transverse. Any region with a voltage of ≤0.05 mV was defined as a scar. All intracardiac electrograms were recorded and analyzed in a recording system (ProLab, BARD Electrophysiology, Lowell, MA, USA) and filtered with 30 to 300 Hz. The variation in cycle length of tachycardia (TCL) was calculated by the maximum minus minimum of TCL, which was counted continuously for 1 min after AT had stabilized for at least 5 min. The fluctuations in TCL were assessed by using the ratio of TCL variation to the mean minimum value of TCL.

Radiofrequency energy was applied in a temperature-control mode with a cut-off of 50°C and an irrigation rate of 20 ml/min. At the posterior wall or in the CS, energy was limited to a maximum of 30 or 25 W, respectively.

### Classifications of Non-Macroreentrant AT and Localization of AT

**Figure 2:**
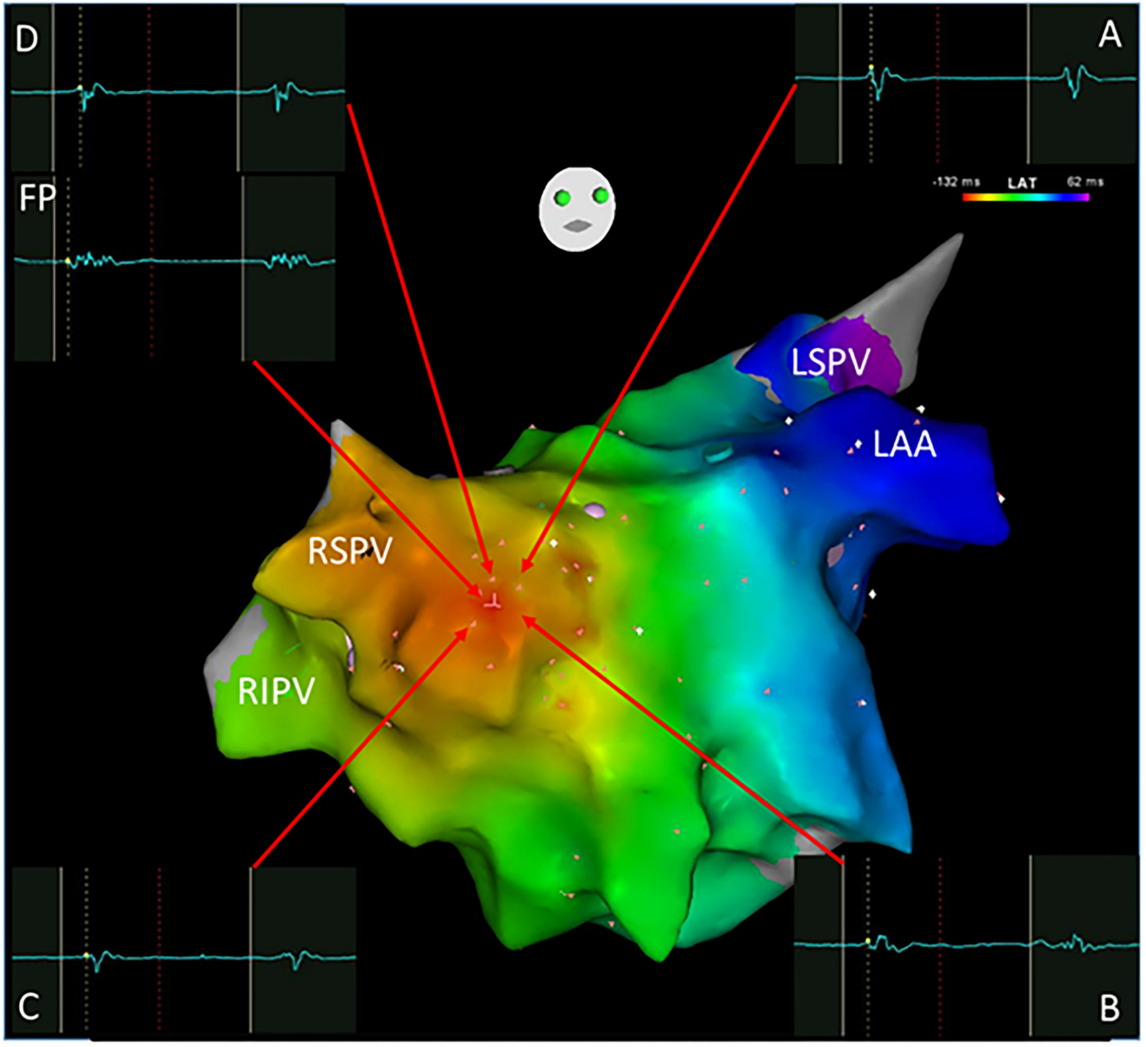
A focal atrial tachycardia located in front of the right superior pulmonary vein with a radiation activation pattern was mapped using the Carto system. The earliest activation point was shown with an FP (-132 ms) as the ablation target. Cross-mapping was employed with local activation time at sites A, B, C, and D for -120,-122,-122, and -122 ms, respectively. FP, fractionated potential; LAA, left atrial appendage; LSPV, left superior pulmonary vein; RSPV, right superior pulmonary vein; RIPV, right inferior pulmonary vein.

**Figure 3:**
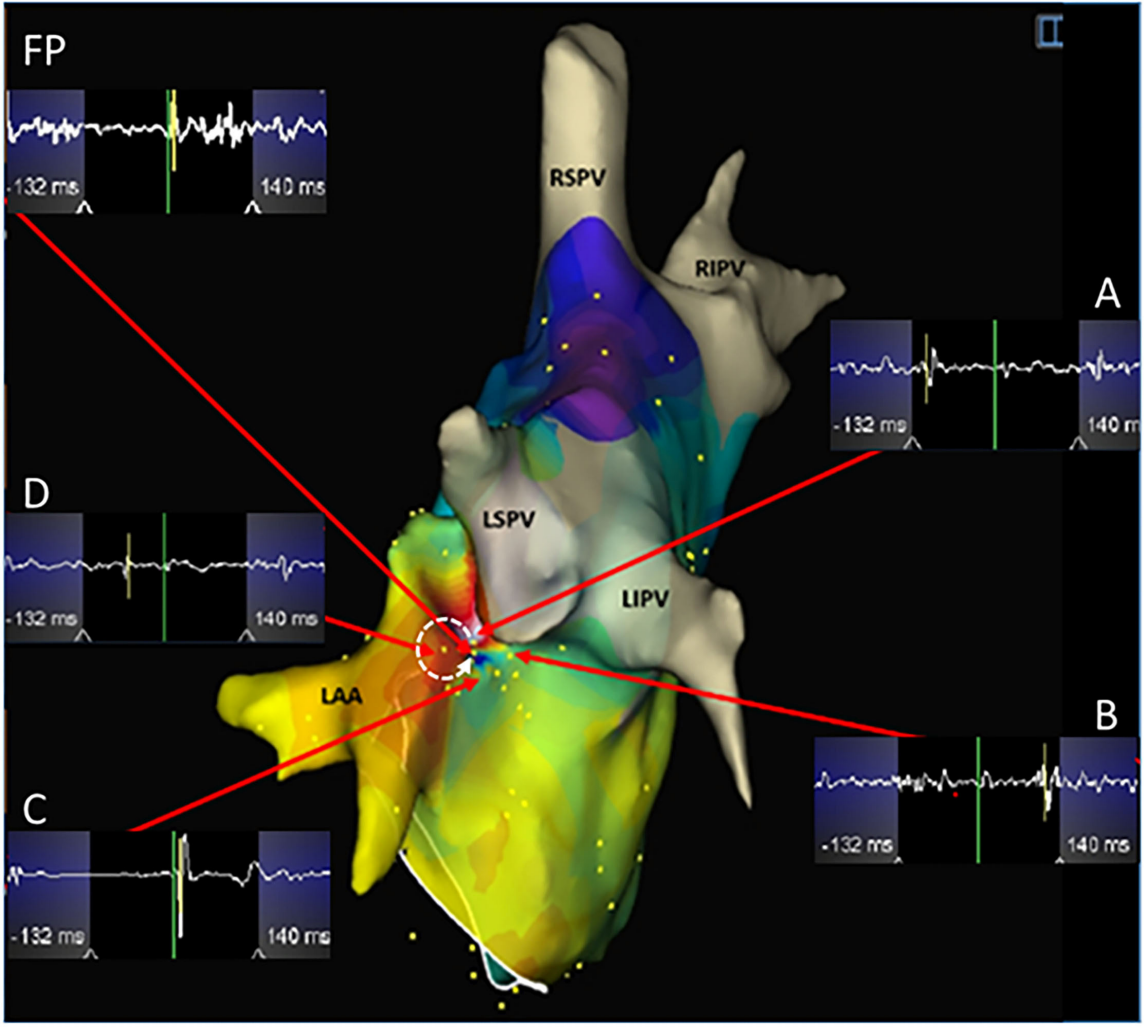
A microreentrant atrial tachycardia located between the left atrial appendage and the left superior pulmonary vein was shown by the EnSite NavX system. A whirlpool-like reentry around a tiny core was displayed with the earliest (white) connected to the latest activation (purple) in an area with a diameter of <10 mm. A wide fractionated potential was recorded connecting the earliest and the latest activation sites. Cross-mapping was used and the local activation time at sites A, B, C, and D measured at -110, 112, 14, and -56 ms, respectively. The dotted arrow shows the activation direction from A to D and further to C and B in sequence. Ablation targeting the FP terminated the atrial tachycardia. LIPV, left inferior pulmonary vein. Abbreviations as shown in Figures 1, 2.

**Figure 4:**
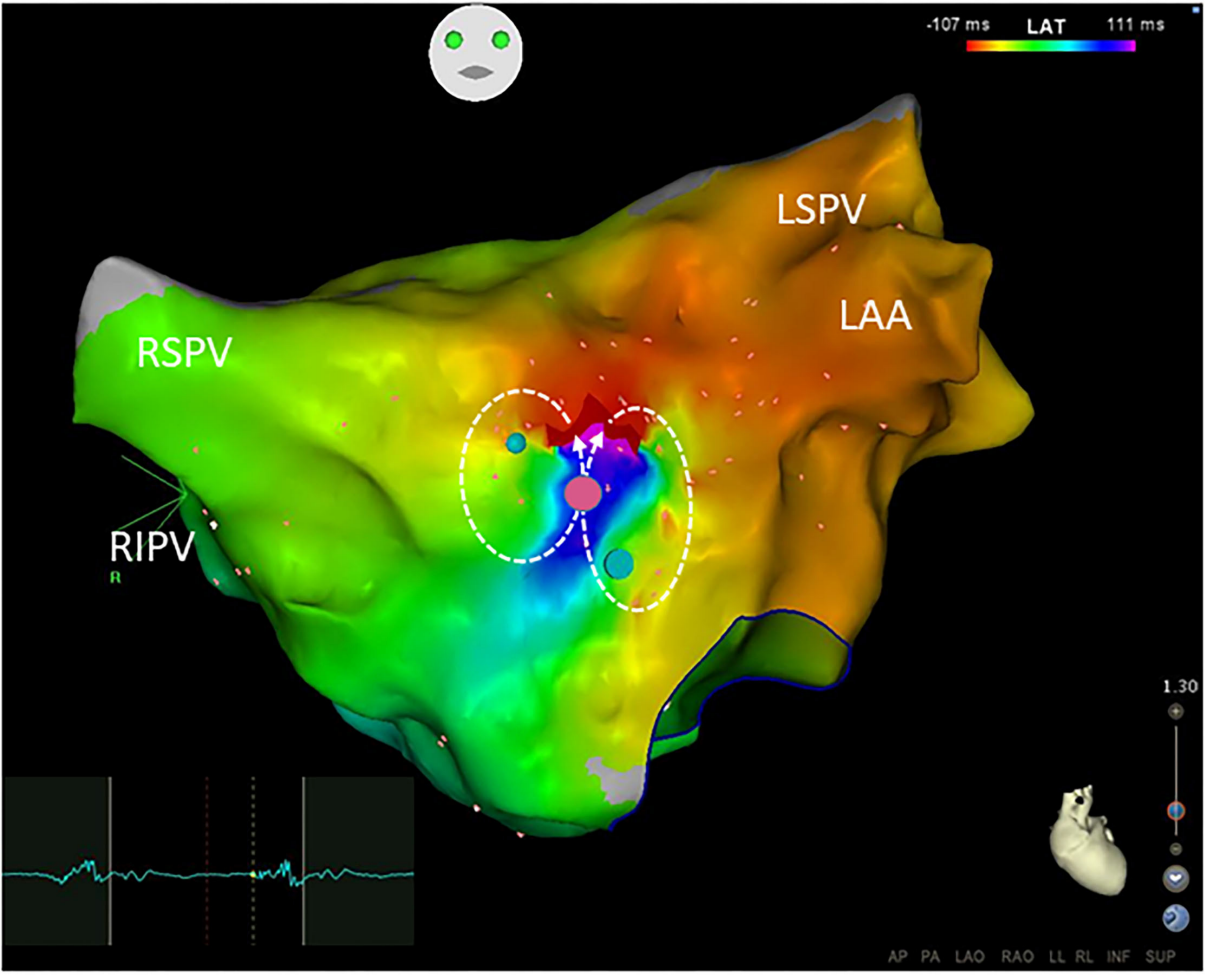
Small-loop-reentrant atrial tachycardia with a figure-of-eight pattern was demonstrated by the Carto system. The reentrant circuits located at the anterior wall of the left atrium were within an area with a diameter of <30 mm but >10 mm. The phenomenon of early (red) meeting late (purple) wavefront was shown in the activation map. A narrow isthmus area with an FP (electrogram on the left) at a pink dot was shown and confined by small regions of the scar. Blue dots define double potentials and dotted arrows show the activation directions. Ablation targeting on the FP terminated the atrial tachycardia. Abbreviations as shown in Figures 1, 2.

According to the global activation patterns, we classified nATs into three types: focal AT (FAT, a radial mode of conduction from the earliest activation site was displayed; Figure 2), microreentrant AT (MAT, a centrifugal mode of reentrant activation around a tiny core without any scar was shown in an area with a diameter of ≤10 mm; Figure 3), and small-loop-reentrant AT (SAT, a centrifugal mode of reentrant activation around a defined scar was presented in an area with a diameter of >10 mm, but ≤30 mm; Figure 4). We localized the site of AT according to the anatomical structure, including the pulmonary vein area (inside or within the antra ≤10 mm from the pulmonary vein ostia), the anterior, posterior, lateral, inferior way, roof, and septum of the LA, CS, and RA (10).

### Follow-Up

After discharge, all patients were regularly followed up in an outpatient clinic. Holter was performed for every patient after 3, 6, and 12 months. If a patient experienced symptoms suggestive of recurrence, a surface electrocardiogram and Holter would be taken at any time.

### Statistical Analysis

Continuous variables were reported as mean ± SD and compared by a one-way ANOVA with a Tukey *post-hoc* test. Categorical variables were reported as absolute or relative frequency and compared by using the chi-square test or Fisher's exact test. All analyses were conducted with the use of SPSS Statistics 25.0 (IBM, Armonk, NY, USA). A *p*-value of < 0.05 was considered statistically significant.

## Results

Thirty-seven patients (31 men and mean age 62 ± 9 years) were included in this study according to the criteria. Coronary artery disease was observed in 3 patients, hypertension in 13, hypertrophic cardiomyopathy in 1, and sick sinus syndrome in 1. The mean left ventricular ejection fraction was 51 ± 6%, and the mean antero-posterior dimension of LA was 42 ± 4 mm. An average of 2 ± 1 ablation procedures for AF were performed on the patients before the AT ablation, including 16 paroxysmal, 7 persistent, and 14 longstanding persistent AF.

In total, 44 nATs were treated, while 31 macroreentrant ATs in 37 patients were ablated during the same procedure. Mapping with CARTO or EnSite NavX system was performed in 15 and 22 patients, respectively, with mean mapping points of 247 ± 36. The mean TCL of these 44 nATs was 316 ± 89 ms (range 208–547 ms). Seventeen ATs were localized within the pulmonary vein areas, 21 in the LA (4 anterior, 5 lateral, 2 inferior, 2 roof, and 8 septum), and 6 in the RA. Compared with the earlier ablation procedures, only 6 ATs were not localized in proximity to the previously ablated areas, including 3 in the anterior LA, 2 in the superior vena cava, and 1 in the RA. Entrainment was performed in 33 ATs, and a postpacing interval of ≤30 ms longer than the TCL was demonstrated in 19 ATs. In the remaining 14 ATs, no postpacing interval of ≤30 ms longer than the TCL was observed in 12 ATs, and arrhythmia changed after pacing in 2 ATs.

All clinical information and electrophysiological data for three different ATs are listed in Tables 1 and 2. There were no differences among FAT, MAT, and SAT regarding the mode of AT initiation, earlier ablation strategies, TCL and its fluctuation. FP was found at a successful ablation target in only 3 out of 12 FATs but all MATs and SATs. Compared to MAT and SAT, mappable TCL was shorter and the FP duration ratio to TCL in the FAT group was lower (*p* < 0.05). Compared to MAT, the ratio of FP duration to TCL was shorter, and more ablation applications were needed to terminate the AT in SAT group (*p* < 0.05). No statistically significant difference was found with respect to FP amplitude (*p* > 0.05).

**Table 1:** Clinical data of different atrial tachycardias.

	**FAT group (*n* = 12)**	**MAT group (*n* = 14)**	**SAT group (*n* = 18)**
**The mode of AT initiation**			
Spontaneous	3	8	7
Induced	8	5	9
Converted from another arrhythmia	1	1	2
**Prior strategies for AF ablation**			
PVI	3	1	1
PVI+CFAE	1	5	5
PVI+lines	6	5	9
PVI+CFAE+lines	2	3	3
**Termination pattern**			
Restore sinus rhythm	12	12	14
Converted to another atrial tachycardia	0	2	4

*AF, atrial fibrillation; CFAE, complex fractionated atrial electrogram; FAT, focal atrial tachycardia; MAT, micro-reentrant atrial tachycardia; PVI, pulmonary vein isolation; SAT, small-loop-reentrant atrial tachycardia*.

**Table 2:** Comparison of electrophysiological data between different atrial tachycardia groups.

	**FAT group (*n* = 12)**	**MAT group (*n* = 14)**	**SAT group (*n* = 18)**	***P* value**
TCL (ms)	323 ± 86 (227–493)	301 ± 93 (208–547)	323 ± 90 (221–500)	0.764
Fluctuation of TCL (%)	2 ± 1 (1–4)	4 ± 2 (1–7)	3 ± 2 (1–8)	0.166
Ratio of mappable CL vs. TCL (%)	63 ± 22 (27–98)	90 ± 9^†^ (64–100)	89 ± 8^†^ (73–100)	**0.009**
Duration of FP (ms)	89 ± 17^*^ (78–108)	161 ± 69 (101–352)	135 ± 48 (60–240)	0.099
Ratio of FP duration versus TCL (%)	28 ± 7^*^ (20–35)	53 ± 11^†^ (34–74)	42 ± 14^†^^‡^ (19–68)	**0.013**
Amplitude of FP (mV)	0.11 ± 0.04^*^ (0.07–0.15)	0.09 ± 0.04 (0.04–0.19)	0.10 ± 0.06 (0.05–0.28)	0.755
Duration of ablation for AT termination (s)	167 ± 183 (2–558)	111 ± 125 (5–472)	313 ± 298^‡^ (12–945)	**0.045**

* *FP was observed in only three out of 12 patients*.

† *p < 0.05 compared with FAT*;

‡ *p < 0.05 compared with MAT; AT, atrial tachycardia; TCL, atrial tachycardia cycle length; FAT, focal atrial tachycardia; FP, fractionated potential; MAT, micro-reentrant atrial tachycardia; SAT, small-loop-reentrant atrial tachycardia. The bold values are < 0.05 meaning significantly different*.

**Figure 5:**
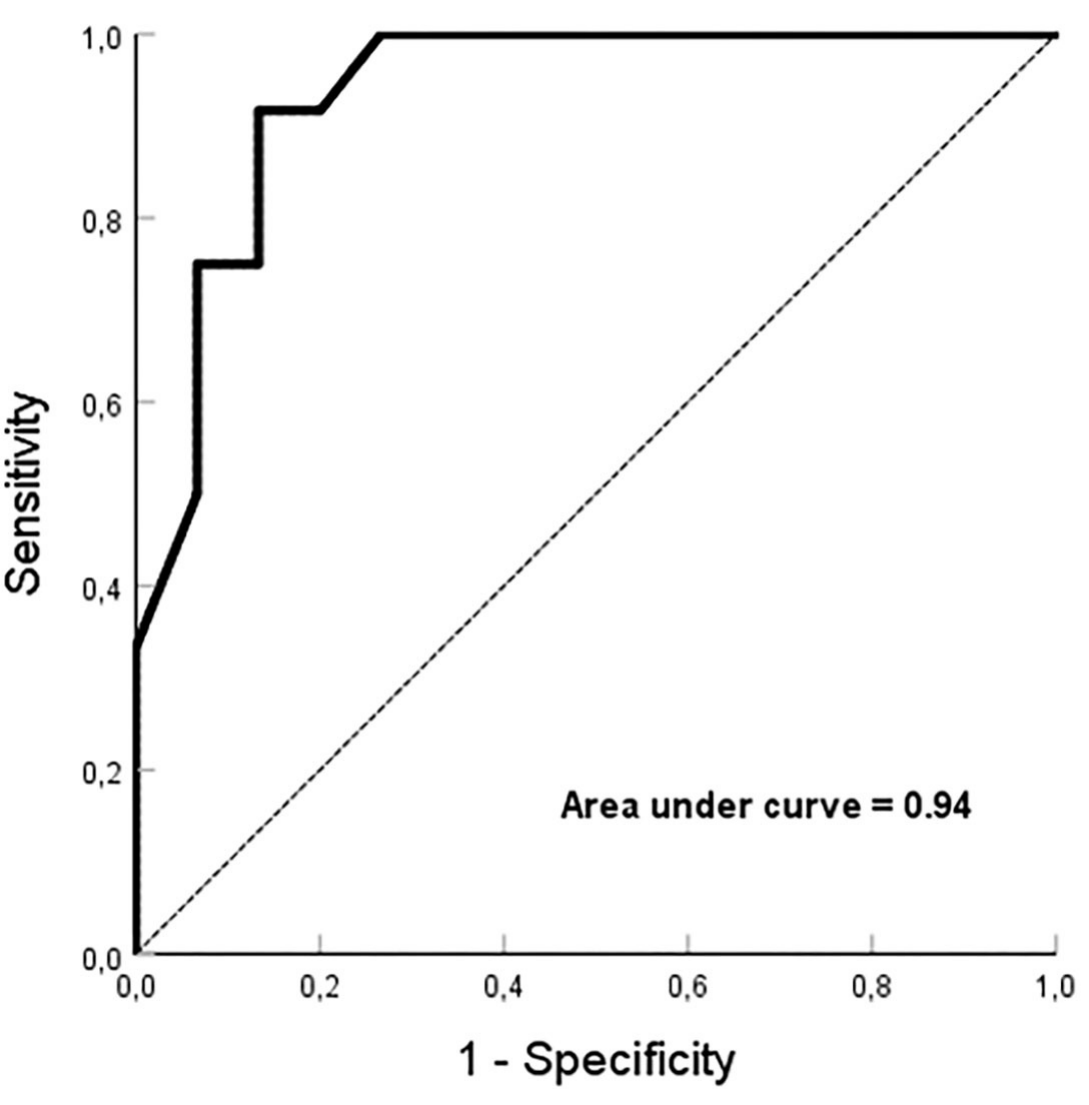
Receiver Operating Characteristic curve analysis. The sum of ≥60% for time-difference ratios in both the longitudinal and transverse directions, is employed as a cut-off value for the fractionated potentials responsible for microreentrant atrial tachycardia with an area under the curve of 0.94.

When cross-mapping was used to differentiate focal sources and bystander FPs, no difference was found in the time difference between the longitudinal and transverse directions in the FAT group, but great differences in the MAT (*p* < 0.01) and bystander groups (*p* < 0.01). Compared to the bystander, trans-potential time differences in both directions were longer in the culprit FP for MAT (*p* < 0.01, Table 3). After Receiver Operating Characteristic curve analysis using different parameters, we regard it as best to adopt the sum of time-difference ratios in both the longitudinal and transverse directions, with ≥60% as a cut-off value for the FPs responsible for MAT with a sensitivity of 92%, specificity of 87%, and area under the curve of 0.94 (Figure 5). The positive predictive value, negative predictive value, and likelihood were 0.87, 0.81, and 6.88, respectively.

**Table 3:** Cross-mapping measurement around fractionated potentials with different roles.

	**Culprit FP for FAT (*n* = 3)**	**Culprit FP for MAT (*n* = 14)**	**Bystander FP (*n* = 17)**
**Trans-potential measurement, longitudinal** Time difference (ms) Time-difference ratio (%)	10 ± 2 4 ± 1	183 ± 90 60 ± 20	71 ± 57^‡^ 22 ± 21^‡^
**Trans-potential measurement, transverse** Time difference (ms) Time-difference ratio (%)	5 ± 1 2 ± 1	107 ± 73^*^ 35 ± 23^†^	15 ± 15^†^^‡^ 6 ± 5^†^^‡^
**TCL (ms)**	285 ± 52	301 ± 93	355 ± 118

* *p < 0.05*,

† *p < 0.01, compared to the longitudinal direction*.

‡ *p > 0.001, compared to the MAT group. Time-difference ratio: the ratio of the activation-time difference to atrial tachycardia cycle length (TCL). Abbreviation as in Tables 1, 2. See also Figure 1*.

There were no periprocedural or delayed postprocedural complications, including thromboembolism, in any patient. After a long-term follow-up period of 69 ± 20 months, six patients experienced AF recurrence, and four had episodes of AT, in which macroreentry was demonstrated during a repeat procedure, while nAT was not documented in any patient. One patient with AT recurrence died of cancer during the follow-up period.

## Discussion

In this study, the electrophysiological characteristics of nAT, especially of FPs, were demonstrated systematically and a new method of cross-mapping was introduced to clarify the roles of FPs.

The ATs following AF ablation usually include the mechanisms of macroreentry and non-macroreentry. Macroreentrant AT is frequently present and can be quickly identified by using three-dimensional mapping combined with entrainment pacing maneuvers (14), while non-macroreentrant AT is more difficult to identify due to its variability, individuality, and diversity. In this study, we focused primarily on three-dimensional activation mapping and the new cross-mapping with weighting less the entrainment pacing maneuvers. The reasons for doing so were based on the difficulties in clinical practice such as (i) rapid stimulation can change the tachycardia to a different arrhythmia (e.g., AF) if the TCL is very short; (ii) stimulation can be hard to capture in a locally injured myocardium (with low voltage) after the previous ablation; (iii) the TCL can be variable, making interpretation of entrainment difficult.

Compared to FAT or SAT, it could be even more challenging to recognize the MAT even though three-dimensional mapping was employed. In our series, 7 out of 14 MATs were differentiated by entrainment pacing. However, only one of them was verified by the entrainment criterion of postpacing interval-TCL of ≤30 ms. The key point of recognizing MAT may be to interpret and identify the essential FPs which represent the fundamentally slow conduction areas for maintenance of MAT. It has been shown that FP is a prerequisite for some of the reentrant arrhythmias (15). Our results demonstrate that FAT could be simply separated from the other nATs based on the short duration of FP and mappable TCL. Although there were no statistically significant differences between MAT and SAT regarding the FP duration, the ratio of FP duration to TCL may separate these two groups. The FP covered more than half of the TCL, which suggests that local slow conduction might be taking place. In this setting, a high-density mapping may be critical. Figure 3 shows that both the earliest and latest activation sites can be localized within a distance of 5 mm from each other. Reducing the size of the mapping catheter electrodes and of the distance between electrodes can certainly increase the resolution of mapping and further increase the chances of recognizing SAT and MAT.

FP may not always present a critical part of the reentrant arrhythmia but is sometimes just a bystander that is passively activated (16, 17). It could be time-consuming if several FPs need to be checked. As a supplement, we introduced a simple method of cross-mapping around the FP to differentiate the bystander FP from the culprit in MAT, and from FAT as well (Figure 1). After four points with local activation time around an FP had been recorded, the ratios of time difference to TCL between two opposite points could be easily obtained and further different nAT or bystander FP could be quickly recognized by using the cutoff sum values of the time-difference ratio in both directions (<10% for FAT; ≥60% for MAT). This finding can be shared with the concept of the new multiple-electrode mapping catheter (18) and potentially developed into new application software. This method can also play an important role when entrainment is impossibly or unsuccessfully performed, or as an additional approach to confirm a diagnosis even while a three-dimensional mapping is employed. Besides being the cause of myocardial degeneration, FP is probably an iatrogenic consequence of non-transmural injury after ablation (17). In the present study, FPs were found on the target sites in 34 out of 44 ATs, which were located in or adjacent to the previous ablation sites. Moreover, there was no difference regarding earlier ablation strategies among the three groups. Thus, a non-transmural injury, no matter what strategy was involved, might create an essential substrate for nAT (19). This indicated that extensive ablation in the atria other than pulmonary vein isolation must be carefully considered.

Being consistent with previous studies, this study has demonstrated that the pulmonary veins, septum, LA appendage, and CS were the common harbors for nAT after AF ablation (20–22). In some patients, the ridges between the pulmonary veins and LA or appendage are steep, which may make the catheter unstable. For this reason, ablation is often performed in these areas with a low contact force (23). The thickness of the myocardium is another factor. The superior parts of the left lateral ridge and septum are the thickest areas in the LA (24, 25). Thus, transmural lesions in these areas are difficult to achieve through endocardial ablation. Furthermore, heterogeneous fiber orientation also provides a milieu for the formation of conduction block/slow conduction and initiation of reentry (26). In CS, complicated connections within the musculature provide an important substrate for reentrant AT (27). Unlike previous studies, the ratio of mappable cycle length to TCL in the FAT group was significantly lower than that in the other groups, but even so, we found a mean of 63%. This may be a characteristic difference from idiopathic focal AT. Although the fluctuation of TCL >15% was reported as a characteristic of focal AT (28), only a mean 2–4% fluctuation was found in our study. The statistically significant difference in ablation duration between the MAT and SAT groups provided further evidence of the tiny circuit size involved in MAT.

## Limitations

This was a retrospective study with a relatively small number of patients. In most cases, multiple ATs coexisted in a single patient post AF ablation. Therefore, it was difficult to select a homogeneous patient population for each type of AT. Notably, high-resolution mapping catheters were not available at the time when the study was performed. Entrainment pacing maneuvers were not obtained in all ATs for various reasons as indicated in the Discussion. These two factors may influence the interpretation and analysis. However, all diagnoses were made based on global mapping in the atria, and not dependent only on a local electrogram. Furthermore, all mappings were verified by the successful ablation outcomes. Additionally, we performed a detailed mapping with help of cross-mapping which could overcome some flaws. It is important to note that interpretation of cross-mapping can be affected in cases where there are existing lines of block in a contiguous area. The cross-mapping method needs to be further confirmed by prospective and randomized studies.

## Conclusion

In this study, characteristic FPs were observed on targets in most nATs following previous AF ablation. The ratio of FP duration to TCL may be of help for differentiation. A simple method of cross-mapping could be employed to clarify the roles of FPs.

## Data Availability Statement

The data analyzed in this study is subject to the following licenses/restrictions: Data related to patients' information cannot be published publicly. Data of this study are saved in the lab and patients' medical recordings. Requests to access these datasets should be directed to eivind.solheim@helse-bergen.no.

## Ethics Statement

The studies involving human participants were reviewed and approved by Regional Ethics Committee of Western Norway. Written informed consent for participation was not required for this study in accordance with the national legislation and the institutional requirements.

## Author Contributions

Y-CW, L-BS, and JC: conception of the study. Y-CW, L-BS, S-YC, and JC: design and interpretation of data, as well as revising of the manuscript. Y-CW, L-BS, S-YC, ES, PS, and JC have significantly contributed to this manuscript, data collection, data analysis, and drafting of the manuscript. All authors have read and approved submission of the manuscript and the manuscript has not been published and is not being considered for publication elsewhere in whole or part in any language except as an abstract. All authors contributed to the article and approved the submitted version.

## Funding

The author L-BS was supported by Helse Vest, Norway, Research Fellowship, and S-YC was supported by the Research Council of Norway.

## Conflict of Interest

JC serves as a consultant for Biosense Webster, Johnson & Johnson. The remaining authors declare that the research was conducted in the absence of any commercial or financial relationships that could be construed as a potential conflict of interest.

## Publisher's Note

All claims expressed in this article are solely those of the authors and do not necessarily represent those of their affiliated organizations, or those of the publisher, the editors and the reviewers. Any product that may be evaluated in this article, or claim that may be made by its manufacturer, is not guaranteed or endorsed by the publisher.
